# Aseptic Meningitis Epidemic during a West Nile Virus Avian Epizootic

**DOI:** 10.3201/eid0909.030068

**Published:** 2003-09

**Authors:** Kathleen G. Julian, James A. Mullins, Annette Olin, Heather Peters, W. Allan Nix, M. Steven Oberste, Judith C. Lovchik, Amy Bergmann, Ross J. Brechner, Robert A. Myers, Anthony A. Marfin, Grant L. Campbell

**Affiliations:** * Centers for Disease Control and Prevention, Atlanta, Georgia, USA; † Centers for Disease Control and Prevention, Atlanta, Georgia, USA; ‡ St. Matthew’s School of Medicine, Grand Caymans, British West Indies; § Maryland Department of Health and Mental Hygiene, Baltimore, Maryland, USA; ¶ University of Maryland Medical Center, Baltimore, Maryland, USA

**Keywords:** Meningitis, aseptic, epidemiology, enterovirus infections, echovirus infections, West Nile fever, disease outbreaks, communicable diseases, emerging, population surveillance

## Abstract

While enteroviruses have been the most commonly identified cause of aseptic meningitis in the United States, the role of the emerging, neurotropic West Nile virus (WNV) is not clear. In summer 2001, an aseptic meningitis epidemic occurring in an area of a WNV epizootic in Baltimore, Maryland, was investigated to determine the relative contributions of WNV and enteroviruses. A total of 113 aseptic meningitis cases with onsets from June 1 to September 30, 2001, were identified at six hospitals. WNV immunoglobulin M tests were negative for 69 patients with available specimens; however, 43 (61%) of 70 patients tested enterovirus-positive by viral culture or polymerase chain reaction. Most (76%) of the serotyped enteroviruses were echoviruses 13 and 18. Enteroviruses, including previously rarely detected echoviruses, likely caused most aseptic meningitis cases in this epidemic. No WNV meningitis cases were identified. Even in areas of WNV epizootics, enteroviruses continue to be important causative agents of aseptic meningitis.

When national surveillance for aseptic meningitis was conducted in the United States, the Centers for Disease Control and Prevention (CDC) received reports of 7,000 to 14,000 cases of aseptic meningitis per year from 1984 to 1994 (*1*). Enteroviruses are the leading identifiable cause of aseptic meningitis in children and adults, particularly in summer and autumn (*2*). In smaller proportions, mumps virus (primarily in studies before 1980), herpesviruses, lymphocytic choriomeningitis virus, arboviruses, *Leptospira,* and many other viral and nonviral agents have been identified in etiologic studies of aseptic meningitis in the United States (*3*,*4*). However, the epidemiology of aseptic meningitis is not static and, with the appearance of emerging infectious agents such as West Nile virus (WNV), warrants periodic reevaluation.

WNV infection is usually asymptomatic but may cause a wide range of syndromes including nonspecific febrile illness, meningitis, and encephalitis. In recent WNV epidemics in which neurologic manifestations were prominent (Romania, 1996 [*5*]; United States, 1999–2000 [*6**,**7*]; and Israel, 2000 [*8*]), meningitis was the primary manifestation in 16% to 40% of hospitalized patients with WNV disease. However, because WNV meningitis has nonspecific clinical manifestations and requires laboratory testing for a definitive diagnosis, case ascertainment and testing practices can affect the number of cases diagnosed. Because WNV testing in U.S. surveillance programs has focused on patients with encephalitis of undetermined cause (*9*), the role of WNV as a cause of aseptic meningitis in the United States is not clear.

A 2001 investigation in Baltimore provided an opportunity to evaluate the role of WNV in the epidemiology of aseptic meningitis and to assess WNV surveillance. From Baltimore City and County, 118 cases of aseptic meningitis with onsets from June 1 to September 30, 2001, were reported to Maryland’s Department of Health and Mental Hygiene (DHMH), compared to an average of 39 cases during the same summer season in 1997*–*2000. Approximately 95% of these 2001 cases were reported without known cause. Simultaneously, an intense WNV epizootic among birds was detected in the Baltimore area (288 WNV-infected dead birds and 14 WNV-infected mosquito pools were collected before September 30, 2001). Early in the summer, nearly 100% of dead crows collected from some sections of Baltimore City tested positive for WNV. When the investigation of aseptic meningitis was initiated in mid-September, one case of human WNV encephalitis had been reported from Baltimore. The investigation’s objectives included 1) identification of the predominant cause(s) of the aseptic meningitis epidemic, emphasizing the relative contributions of WNV and enteroviruses and 2) evaluation of hospital-based–WNV surveillance of patients with aseptic meningitis, including strategies used for diagnostic testing.

## Methods

### Discharge Diagnoses Code Review

To confirm an increase in aseptic meningitis cases by a method independent of case reporting to DHMH, a discharge diagnosis code review was conducted at the six investigation hospitals. Included were patients with aseptic meningitis (including International Classification of Diseases [ICD]-9-CM codes 047.0, 047.1, 047.8, 047.9, 049.0, 049.1, 053.0, 054.72, 072.1) who were evaluated from June 1 to September 30, 1998*–*2001.

### Case Definitions

The investigation was conducted at six hospitals that collectively reported to DHMH 76% of the 118 aseptic meningitis cases from Baltimore City and County. A case of aseptic meningitis was defined as an illness with onset from June 1 to September 30, 2001; cerebrospinal fluid (CSF) cell count of >5 leukocytes per milliliter; negative CSF bacterial cultures; and no physician or laboratory documentation of bacterial, fungal, or parasitic central nervous system (CNS) disease, cerebral hemorrhage, carcinomatous meningitis, or cerebral vasculitis. Neonates who developed CSF abnormalities before first hospital discharge were excluded, as were persons with a physician-documented diagnosis of encephalitis, confusion, or obtundation. A case of enteroviral meningitis was defined as an illness meeting the criteria for aseptic meningitis and, in addition, a positive enterovirus culture of a CSF, rectal swab, or nasopharyngeal specimen, or a positive enterovirus polymerase chain reaction (PCR) test of a CSF specimen. A case of WNV meningitis was defined as an illness meeting the criteria for aseptic meningitis and, in addition, WNV immunoglobulin (Ig) M detected in a CSF specimen by enzyme-linked immunosorbent assay (ELISA), a greater than-four-fold rise of WNV-neutralizing antibodies in acute- and convalescent-phase serum specimens, or WNV nucleic acid detected in a CSF specimen by PCR. The investigation was limited to persons living in Baltimore City or County who were evaluated at one of the six investigation hospitals.

### Case Ascertainment

Cases reported to DHMH as the code “viral meningitis” were reviewed. Depending on the resources of each hospital, additional cases were identified by computerized queries for test results of >5–10 leukocytes per milliliter in CSF, and by review of hospital discharge diagnoses codes. For each case, a standardized form was used to abstract clinical information from the medical record.

### Acute-Phase and Convalescent-Phase Specimens and Interviews

Acute-phase (<8 days after illness onset) CSF, serum, rectal swab, and nasopharyngeal specimens were collected if the ordered by the patients’ physicians. Specimens were stored at hospital, DHMH, or private reference laboratories at temperatures ranging from 4°C to –70°C. During home visits to consenting patients >12 years of age who had had aseptic meningitis of unknown cause, convalescent-phase (>7 days after illness onset) blood specimens were collected and a standardized questionnaire was administered to characterize symptoms and duration of illness.

### Diagnostic Testing

Hospitals performed routine cell counts, chemistries, and bacterial cultures of CSF for patients with sufficient specimen quantity. Some patients’ physicians ordered additional tests. These tests commonly included, for CSF specimens, latex agglutination screening tests for bacterial antigens, culture or PCR tests for enteroviruses or herpesviruses, and culture for fungi; for CSF or serum specimens, *Borrelia burgdorferi* antibody, Venereal Disease Research Laboratory slide test, and cryptococcal antigen tests; and for nasopharyngeal and rectal swab specimens, culture for enteroviruses.

For available specimens from patients with aseptic meningitis of unknown cause, arbovirus serologic testing was performed at DHMH laboratories, and WNV PCR tests were completed at DHMH laboratories or the Division of Vector-Borne Infectious Diseases, CDC. CSF specimens were tested by ELISA for IgM antibodies to WNV and by TaqMan reverse-transcriptase (RT-) PCR tests for WNV (*10*,*11*). Serum specimens were tested at DHMH laboratories by ELISA for IgM antibodies to WNV, and by immunofluorescence assay (IFA) for IgM and IgG antibodies to La Crosse, St. Louis encephalitis, eastern equine encephalomyelitis, and western equine encephalomyelitis viruses.

Available CSF and rectal swab specimens from patients with aseptic meningitis of unknown cause were tested by enterovirus culture at the Respiratory and Enteric Viruses Branch, CDC. Three cell lines were used at CDC for enterovirus culture: RD (human rhabdomyosarcoma), HELF (human embryonic lung fibroblast), and LLC-MK2 (monkey kidney). Isolates were serotyped by RT-PCR amplification and subsequent sequencing of an approximately 320-nt segment of the VP1 enterovirus gene (*12*). When available, enteroviruses already isolated by hospital laboratories from CSF, nasopharyngeal, or rectal swab specimens were serotyped by CDC.

### Evaluation of Aseptic Meningitis and WNV Surveillance

To evaluate strategies used to diagnose WNV meningitis in Maryland, reporting and testing policies were reviewed. Information was obtained from DHMH case-reports, surveillance plans and summaries, and laboratory tests of investigation patients.

## Results

### Confirmation of an Epidemic by Discharge Diagnosis Review

**Figure 1:**
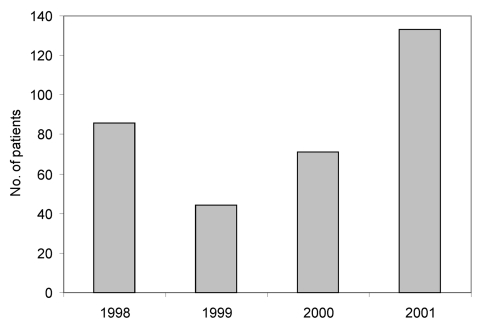
Residents of Baltimore City and County evaluated at six hospitals and assigned aseptic meningitis ICD-9-CM discharge diagnosis codes^a^ during June 1 – September 30, 1998-2001. ^a^ If during one season a patient had >1 discharge diagnosis codes for aseptic meningitis, the patient was only counted once.

Each summer season (June 1–September 30) of 1998–2000, an average of 67 Baltimore residents were evaluated at the six investigation hospitals and assigned an aseptic meningitis ICD-9-CM code; in the 2001 season, 133 persons were evaluated, a 99% increase above the 1998–2000 seasonal average (Figure 1). At on of the investigation hospitals, the specificity of ICD-9-CM codes was assessed. A medical record review showed that 23 (96%) of 24 cases identified by ICD-9-CM codes met the investigation’s case definition of aseptic meningitis.

### Cases

**Figure 2:**
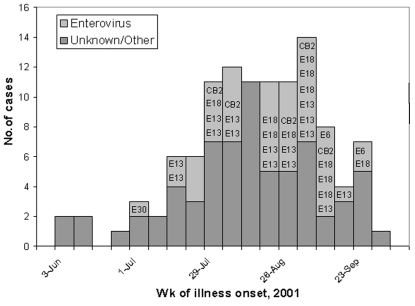
Aseptic meningitis cases* by week of illness onset, June 1–September 30, 2001, identified at six hospitals, Baltimore, Maryland. *N=112 (illness onset date missing for one patient); Coxsackievirus B2 = “CB2”; Echovirus 6 = “E6”, Echovirus 13 = “E13”; Echovirus 18 = “E18”; Echovirus 30 = “E30”

At the six investigation hospitals, 113 aseptic meningitis patients were identified with illness onsets from June 1 to September 30, 2001. By the week of illness onset, the number of cases peaked in late August and early September (Figure 2). The median patient age was 18 years (range 1 week*–*74 years of age), and 56% of patients were male. Seventy-eight percent of patients were medically evaluated within the first 3 days after illness onset. Of the 110 patients with available information, the median duration of hospitalization was 2 days (range 0*–*11 days). No fatalities occurred during hospitalization nor were any subsequently reported to DHMH.

The median CSF leukocytes count was 135/mL (range 7–1,083/mL). Of the 110 patients with available data, 45 (41%) had >50% polymorphonuclear cells in CSF leukocyte count, including 9 (43%) of 21 patients who had CSF collected 4*–*10 days after illness onset. CSF glucose was normal (≥40 mg/dL) in 99% of patients (n=110), and CSF protein was elevated (>50 mg/dL) in 52% (n=111: median 53 mg/dL; range 10*–*215 mg/dL) (Table 1).

**Table 1:** Descriptive summary of aseptic meningitis cases with onsets from June 1–September 30, 2001, identified at six hospitals, Baltimore, Maryland^a^

Cause	Cases	Median age (range)	% <18 y of age	CSF (range)			
				Median leukocyte count/mL (range)	% PMN pre-dominant	Median protein mg/dL (range)	
All enterovirus meningitis cases	43	9 y (1 wk–49 y)	70	178 (10–850)	49	48 (10–215)	
Echovirus 13	15	7 y (1 mo–49 y)	87	132 (11–650)	80	37 (18–97)	
Echovirus 18	11	17 y (2 mo–35 y)	55	173 (12–409)	27	44 (16–215)	
Coxsackievirus B2	5	19 y (1 wk–31 y)	40	250 (45–850)	0	87 (57–120)	
Echovirus 6	2	33 y (31–34 y)	0	330 (130–530)	50	56 (52–59)	
Echovirus 30	1	10 y	1	10	0	18	
Untyped enterovirus	9	9 y (1 mo–20 y)	89	178 (43–850)	56	48 (10–153)	
Herpes simplex virus meningitis cases	2	39 y (29–49 y)	0	246 (136–355)	0	128 (77–179)	
Lyme meningitis case	1	74 y	0	227	0	143	
Cases of undetermined cause	67	25 y (2 wk–67 y)	39	100 (7–1083)	38^b^	53^c^ (19–209)	
Total cases	113	18 y (1 wk–74 y)	50	135 (7–1083)	41^d^	53^e^(10-215)	

^a^CSF, cerebrospinal fluid; PMN, polymorphonuclear cells. ^b^N=64. ^c^N=65. ^d^N=110. ^e^N=111.

On the basis of standardized interviews of 33 patients (median age, 31 years; range 13*–*55 years of age) at the time convalescent-phase blood specimens were obtained, the most commonly reported acute-phase symptoms were headache (100%), fever (85%), and eye pain or sensitivity to light (85%). Illness was sometimes prolonged by persistent fatigue and headaches. The median duration of illness was 18 days (n=32; range 5*–*47 days). Twelve of the patients had not fully recovered by the time of interview; for these patients, the minimum duration of illness was defined as (date of interview) – (date of illness onset). Patients ≥18 years of age reported longer duration of symptoms (n=24; median duration 22 days) than patients 13–17 years of age (n=8; median duration 7 days) (K-W, p=0.001). Clinical findings among different age groups were compared with the Kruskal-Wallis (K-W) test of significance in Epi-Info 6 software.

### WNV Test Results

Of the 69 patients for whom at least one ELISA WNV IgM test was performed on CSF or serum, none tested positive. Of these 69, ELISA WNV IgM testing was performed on both acute- and convalescent-phase specimens for 23 patients, on only acute-phase specimens for 36 patients, and on only convalescent-phase specimens for 10 patients. Arboviral IgM IFAs completed on acute- or convalescent-phase serum specimens from 39 patients were all negative. WNV PCR tests completed on acute-phase CSF specimens from 27 patients were also negative. Acute-phase specimens were collected <8 days after illness onset, and convalescent-phase specimens were collected a median of 40 days after illness onset (range 12–111 days); exact dates were not available for two patients.

### Enterovirus Test Results

Of 70 patients who had at least one test (viral culture or PCR test) performed for enteroviruses, 43 (61%) patients were confirmed to have enteroviral meningitis. Among patients who had at least one enterovirus test performed, the percentage enterovirus-positive was highest in infants and children; however, of 30 tested patients >18 years of age, 13 (43%) were enterovirus-positive (Table 2). Of the 34 cases in which enterovirus serotyping was completed, five serotypes were identified. Echovirus 13 (15 cases) and echovirus 18 (11 cases) together accounted for 76% of the serotyped isolates (Table 1).

**Table 2:** Enterovirus meningitis cases by age group, identified at six investigation hospitals, Baltimore, Maryland, summer 2001

Age group (y)	Aseptic meningitis cases	% Test-positive^a^ for enterovirus (no. test-positive/no. tested for enterovirus)
<1	12	80 (8/10)
1–10	24	94 (15/16)
11–20	29	50 (11/22)
21–30	11	75 (3/4)
31–40	26	38 (5/13)
41–50	5	33 (1/3)
>50	6	0 (0/2)
All	113	61 (43/70)

^a^Thirty-four (79%) of the 43 enterovirus meningitis cases had a positive viral culture or polymerase chain reaction test result of cerebrospinal fluid (CSF) specimens. Seven cases had negative CSF tests and were only diagnosed by positive viral culture of nasopharyngeal or rectal swab specimens. Two additional cases did not have sufficient CSF available for testing and were diagnosed by positive culture of nasopharyngeal or rectal swab specimens.

### Other Diagnoses

Two patients were diagnosed with herpes simplex virus meningitis by culture-positive CSF specimens, and one patient was diagnosed with Lyme meningitis on the basis of clinical signs and symptoms of acute Bell’s palsy and meningitis, and positive serum *B. burgdorferi* antibody by ELISA and Western blot tests.

Sixty-seven (59%) of the 113 patients in the investigation remained undiagnosed; for many, sufficient specimens did not exist for further testing. The median age of these undiagnosed patients was 25 years, and 61% were male. Duration of hospitalization was similar to the cases with known cause. Five of the undiagnosed patients had HIV infection, and another four had a history of prior meningitis. Twenty-seven (40%) of the undiagnosed patients had at least one enterovirus test (culture or PCR) performed. Forty-six (68%) of the undiagnosed patients, including 24 with convalescent-phase specimens collected a median of 44 days after illness onset (exact dates not available for two patients) (range 12–111 days), had at least one WNV IgM ELISA performed. To estimate the number of WNV meningitis cases that could have been missed, we assumed that these 24 patients represented a random sample of the 67 undiagnosed patients, and that WNV infection was ruled out in patients with a negative result from a WNV IgM ELISA performed on a convalescent-phase serum. On the basis of these assumptions, 0% (95% confidence interval 0% to 10%) WNV IgM positivity among the sample suggests that fewer than seven cases of WNV meningitis were missed among the investigation patients.

### Evaluation of Aseptic Meningitis and WNV Surveillance

Human WNV surveillance in Maryland focused on patients with two reportable CNS infections, encephalitis or meningitis, of unknown cause. Of the 113 aseptic meningitis cases identified at the six investigation hospitals, 76 (67%) had been reported to DHMH. Of these 76 aseptic meningitis cases, 71 (93%) were reported without a cause. Because of the urgency to detect WNV epidemics, WNV serologic testing with the first-line test was conducted by DHMH laboratories for patients with CNS infections of unknown cause. WNV testing was first prioritized for patients with encephalitis, and secondarily for hospitalized patients with aseptic meningitis who were >17 years of age (late in the season, this last criteria was expanded to >5 years of age). During 2001, DHMH laboratories conducted WNV testing for 440 patients statewide (including approximately 230 patients reported with aseptic meningitis); 6 patients were diagnosed with WNV disease.

DHMH requested CSF, serum, and convalescent-phase serum specimens for the diagnosis of WNV infection. However, before the investigation, essentially only acute-phase specimens (CSF more frequently than serum) from Baltimore patients were tested by WNV serologic tests; routine collection of convalescent-phase serum specimens was not feasible.

Enterovirus testing was not a component of DHMH aseptic meningitis surveillance. If enterovirus testing was initiated by the hospital, positive results were often not communicated to DHMH laboratories. Of 69 patients for whom at least one WNV IgM test was performed by DHMH, enteroviral meningitis was subsequently diagnosed in 23 (50% of 46 for whom at least one enterovirus culture or PCR test was performed).

## Discussion

Although enhanced WNV surveillance among persons with aseptic meningitis could have been partially responsible for the tripling of Baltimore case-reports of aseptic meningitis during the summer of 2001, trends in discharge diagnosis codes suggest that a true increase in aseptic meningitis cases occurred. Despite a concurrent, intense WNV epizootic among birds, no evidence existed that WNV substantially contributed to the aseptic meningitis epidemic. By routine surveillance, five WNV encephalitis cases but no WNV meningitis cases were ultimately detected in Baltimore in 2001. However, in this setting, the five recognized WNV encephalitis cases did not appear to represent large numbers of undiagnosed WNV meningitis cases. Surveillance conducted in other states has also suggested that intense WNV epizootic activity does not necessarily correlate with large numbers of human WNV CNS infections (*13*).

Instead, multiple enterovirus serotypes likely caused most of the Baltimore aseptic meningitis cases. This finding is consistent with population-based studies (*14*–*16*) and large outbreak investigations (*17*,*18*) of aseptic meningitis occurring during the summer and fall in the United States. However, unlike outbreak investigations of the past few decades, echovirus 13 and echovirus 18 were the most commonly isolated agents in this investigation and might have accounted for the increased number of aseptic meningitis cases in Baltimore. Echovirus 13 was previously rarely detected in the United States. From 1970 to 2000, only 65 of 45,000 enterovirus isolates reported to CDC were echovirus 13 (*19*). Echovirus 18 had been relatively quiescent for over a decade; from 1988 to 2000, only 200 isolates were reported to CDC (20). In a study conducted from 1986 to 1990 in Baltimore hospitals among infants <2 years of age who were hospitalized with aseptic meningitis, only one case of echovirus 13 and two cases of echovirus 18 (among 167 serotyped enterovirus isolates) meningitis were identified (*14*).

In 2001, the previously rarely detected echoviruses 13 and 18 were the two enteroviruses most commonly reported to CDC (*20*). Multiple states reported echovirus 13 in association with aseptic meningitis outbreaks, and Tennessee reported an aseptic meningitis outbreak attributed to both echovirus 13 and 18 (*19*). Where previously rarely detected, echovirus 13 was isolated in association with aseptic meningitis outbreaks in Australia during 2001 (*21*) and in the United Kingdom (*22*) and Germany (*23*) during 2000. Surveillance data have previously demonstrated that in one or two seasons a usually quiescent serotype may cause an outburst of clinical disease superimposed on background, area-endemic enteroviruses (*24*). The worldwide spread of epidemics of clinical enteroviral disease has been documented with other serotypes, including echovirus 9 and enterovirus 70 (*25*).

Limitations of this study should be acknowledged. Physicians may have diagnosed more aseptic meningitis in response to WNV publicity; however, physicians more likely recognized that the risk and discomfort of a lumbar puncture required to diagnose meningitis outweighed the public health interest in identifying an untreatable condition, WNV meningitis. Regarding the investigation, only aseptic meningitis cases that could be rapidly identified at the six Baltimore hospitals were included. Not all patients underwent the same tests in the same laboratories, and the quality of enterovirus testing differed because of variable conditions of specimens. Additional results of other tests performed at reference laboratories might have become available only after the investigation ended. As a result, although any positive WNV test result would likely have been reported, the percentage of enterovirus test-positive cases could be inaccurate. Finally, similar to previous studies of the epidemiology of aseptic meningitis (*2*), 59% of cases remain undiagnosed; another, untested agent may have caused the increased number of aseptic meningitis cases in Baltimore.

The consistent predominance of enteroviruses among the known causes of aseptic meningitis in children and adults versus the relative infrequency of WNV meningitis (even during an intense WNV epizootic) warrants reconsideration of WNV surveillance testing strategies. Most cases of aseptic meningitis were reported to DHMH without a known cause. For these patients, WNV testing was the first priority to enable early detection of WNV epidemics that would warrant additional vector control interventions. By contrast, enterovirus testing was not a component of surveillance among patients with aseptic meningitis. Although no WNV meningitis was identified, >30% of the patients who underwent WNV testing were later determined to have had enteroviral meningitis. Many patients with aseptic meningitis were tested for an apparently rare virus, WNV, before being tested for the common agents, enteroviruses. During nonepidemic years, WNV IgM ELISA may be low yield when performed as a first-line test for aseptic meningitis. By contrast, enterovirus testing likely can identify the cause of a large fraction of aseptic meningitis cases.

The complexities and resource requirements of WNV serologic testing suggest that other testing strategies need to be developed. During 2001, DHMH laboratories conducted WNV testing for 440 patients statewide, often performing multiple tests for each patient; 6 patients were diagnosed with WNV disease. WNV ELISAs require at least 2–3 days to complete. PCR tests for WNV in CSF specimens are more rapid but have poor sensitivity (*26*). Because patients often seek medical care early after illness onset, when WNV antibodies might not be detectable, serologic tests of specimens collected at the time of first symptoms may also have poor sensitivity. Serologic testing of convalescent-phase serum specimens may be the most sensitive method to detect WNV infection. Yet, collecting convalescent-phase specimens can be logistically difficult and, as in Baltimore’s WNV surveillance program, may not be routinely feasible. When collected through primary care physicians, billing issues can be problematic, and each home visit for collection of blood specimens may require several hours of staff time.

An improved laboratory-based surveillance strategy might include a two-stage testing algorithm at hospital or public health laboratories to evaluate patients with aseptic meningitis. The goals would be 1) to judiciously use specimens of limited quantity (especially CSF) to rapidly identify common or treatable causes of aseptic meningitis and 2) to improve the yield of the more complex testing required to diagnose arboviral disease.

As a first stage of testing, multiplex PCR tests have been used to detect enteroviruses, herpes simplex 1 and 2, and varicella zoster (*27*,*28*). Several studies indicate that PCR tests for enteroviruses (*29*) and herpes simplex virus (*30*) are highly specific, can be completed more rapidly (<6 hours required), require less quantity of CSF, and potentially are more sensitive than traditional cell culture. Using PCR tests to identify enterovirus infections in patients with aseptic meningitis can reduce unnecessary ancillary tests and antibiotic or antiviral therapy and allows shortened hospitalizations (*31*). In addition, treatment for enteroviral infection may become available in the near future (*32*).

If no diagnosis is made after completion of screening tests for common or treatable agents and evidence of regional WNV or other arbovirus activity exists, a second stage of testing might include arbovirus IgM ELISA of acute- and convalescent-phase specimens. To rule out WNV disease, WNV IgM and WNV IgG ELISAs may need to be conducted approximately 8–45 days after illness onset (WNV IgG ELISAs may be complicated by cross-reactions that can only be differentiated by logistically difficult plaque reduction neutralization tests) (*10*). The importance of the timing of specimen collection should be clearly communicated to healthcare providers.

The epidemiology of aseptic meningitis and other CNS infections is not fixed and may vary by location; in the same location, the epidemiology may vary by different seasons. For example, while relatively quiescent in most years, WNV has the potential to cause large epidemics in humans. Because WNV disease does not have unique clinical manifestations, adequate laboratory testing is the only way to identify human WNV epidemics. Refinement of laboratory testing strategies for WNV surveillance may help use resources and build broader public health laboratory capacity for arboviral and other CNS infections.
